# Maternal allergy is associated with surface-bound IgE on cord blood basophils

**DOI:** 10.1111/pai.12113

**Published:** 2013-08-27

**Authors:** Adam P Matson, Michelle M Cloutier, Ashish Dhongade, Lynn Puddington, Ektor Rafti

**Affiliations:** 1 Department of Immunology, University of Connecticut Health CenterFarmington, CT, USA; 2 Division of Neonatology, Connecticut Children’s Medical CenterHartford, CT, USA; 3 Asthma Center, Connecticut Children’s Medical CenterHartford, CT, USA

**Keywords:** asthma, basophils, cord blood, dendritic cells, maternal influence

## Abstract

**Background**The cell type(s) mediating the maternal influence on allergic disease in children remain unclear. We set out to define the relationship between maternal allergy and frequencies of cord blood (CB) basophils, and plasmacytoid dendritic cells (pDCs); to characterize surface-bound IgE and FcεRI expressions on these cells; and to investigate the association between maternal and CB serum IgE levels with surface-bound IgE and FcεRI expressions.

**Methods**One hundred and three mother/infant dyads were recruited prenatally, and maternal allergic history was recorded. Maternal blood was collected prior to delivery, and CB was collected after birth. Flow cytometry was used to identify CB basophils and pDCs and to determine surface-bound IgE and FcεRI expressions.

**Results**Frequencies of CB basophils and pDCs were low and not related to maternal history of allergy. Percentages of CB basophils with surface-bound IgE were significantly higher in infants of allergic mothers compared with infants of non-allergic mothers (median, 59.60% vs. 19.70%, p* *= 0.01). IgE on CB basophils correlated with CB IgE levels (r = 0.72, p < 0.0001), but not with maternal IgE levels (r = 0.26, p = 0.06). IgE on CB pDCs was low and not significantly associated with maternal or CB IgE levels. Similarly, FcεRI expression by CB basophils and pDCs was not significantly associated with maternal or CB IgE levels.

**Conclusions**Frequencies of CB basophils and pDCs are not influenced by maternal allergy. CB basophils and pDCs have surface-bound IgE and express FcεRI; however, only IgE on CB basophils appears influenced by maternal allergy.

Maternal allergy is a risk factor for the development of allergic disease in children 1–4; however, the cell types mediating this maternal influence remain poorly understood. Basophils participate in allergic responses by releasing histamine upon cross-linking the immunoglobulin E (IgE) antibodies bound to the high-affinity IgE receptor (FcεRI) 5, 6. Increased numbers of basophils are found in several tissues from individuals with allergic disease 7–9, and Th2-type cytokines produced by activated basophils enhance the ability of CD4^+^ T cells to promote B cell proliferation and immunoglobulin production 10.

Plasmacytoid dendritic cells (pDCs) are potent antigen-presenting cells (APCs) that appear important for host defense against viral infections by the production of type I interferon, particularly at mucosal surfaces 11. Their involvement in allergic disease is suggested by the finding that pDCs from adults with atopy express FcεRI, which is regulated by serum IgE levels 12–14. Other studies report that a deficiency of circulating pDCs at 6–12 months of age is a risk factor for respiratory tract infections and the diagnosis of asthma by 5 yr of life 15. Given the apparent role of basophils and pDCs in the pathogenesis of allergic disease, we sought to determine the influence of maternal allergic status on the frequencies of these cell types in cord blood specimens. In addition, we aimed to define the relationship between surface-bound IgE and FcεRI expressions on these cells relative to maternal allergic status and maternal and CB serum IgE levels.

## Methods

### Study design and participants

Allergic and non-allergic pregnant women receiving care at Hartford Hospital Associated OB/GYN practices (Hartford, CT, USA) were recruited prior to delivery. Recruiting strategy and eligibility criteria are described in the electronic repository (ER).

Pregnant women were divided into two groups based on the absence or presence of allergic disease defined by a physician’s diagnosis of asthma, allergic rhinitis, atopic dermatitis, or food allergy, and associated symptoms (e.g. cough, wheeze, skin rash) within the past 12 months. The study was approved by the Institutional Review Board at Hartford Hospital, and informed written consent was obtained from all prospective mothers and on behalf of their infants.

### Blood collection for measurement of serum antibodies

Upon admission to Labor and Delivery, maternal blood was obtained by venipuncture. Following delivery, CB samples were obtained from the umbilical cord-placenta unit by means of needle puncture of the umbilical cord vein cleansed with alcohol. Total serum IgE concentrations were determined by Phadia (Thermo Fisher Scientific, MI, USA) using the ImmunoCap method. The detection limit for IgE in maternal and CB specimens was 2.00 KU/l and 0.10 KU/l, respectively. As a surrogate marker for maternal blood contamination, sera from CB samples were analyzed for total IgA levels using a commercial ELISA kit (Mabtech Inc., Cincinnati, OH, USA). The detection limit for IgA was 0.50 μg/ml (based on a serum dilution of 1:100), and CB samples containing ≥10 μg/ml IgA were excluded from the study 16.

### Collection and preparation of CB cells

Cord blood was obtained for isolation and cryopreservation of CB peripheral blood mononuclear cells (CBMCs) using a procedure adapted from the Immune Tolerance Network (PBMC CPT v.009) online protocol and a published protocol by Basford et al. 17. Further information is provided in the ER.

### Flow cytometry

Cord blood basophils and pDCs were identified as the CD123^+^high BDCA-2^-^ and CD123^+^high BDCA-2^+^ cells, respectively 18, 19, and surface-bound IgE and FcεRI expressions were quantified relative to an isotype control antibody. Further information is provided in ER.

In some experiments, CBMCs were pre-incubated with 50 μg monoclonal human IgE (HE1) (Bioreclamation LLC., Westbury, NY, USA) or 400 IU WHO IgE preparation (NIBSC, Potters Bar Hertfordshire, United Kingdom) for 1 h at 18–25°C prior to staining. This was carried out to investigate the ability of CB basophils and pDCs to bind exogenous IgE and whether or not bound IgE blocked binding of the FcεRI detection antibody.

To determine the primary outcome of the relationship between maternal allergy and levels of CB basophils and pDCs, CB samples containing a minimum of 200 basophils and 200 pDCs were analyzed. Frequencies of CB basophils and pDCs were expressed as percentages of total CBMCs. To determine secondary outcomes, CB basophils and pDCs were analyzed for surface-bound IgE and FcεRI expressions. Percentages of CB basophils and pDCs identified by flow cytometry with bound IgE or that expressed FcεRI were compared between infants of allergic and non-allergic mothers. In addition, the association between maternal and CB serum IgE levels with surface-bound IgE and FcεRI expressions was investigated.

### Statistical analysis

Statistical analyses were performed using Prism 4 (GraphPad Software, San Diego, CA, USA). For measured variables, statistical comparisons were performed by the nonparametric Mann–Whitney test. Chi-square was used to compare the distribution of weight gain between allergic and non-allergic mothers, and the Fisher’s exact test was applied to compare the remaining categorical variables between groups. Correlation analyses were performed using Pearson’s correlation. Statistical significance was defined as a p-value ≤ 0.05.

## Results

### Demographics and baseline characteristics

The number of mother/infant dyads recruited, enrolled, and excluded is described in Fig. S1. Samples from 14 allergic and 36 non-allergic mother/infant dyads contained sufficient amounts of CB basophils and pDCs to investigate the primary and secondary outcomes. Baseline characteristics of these mothers and infants are shown in Table 1. For these characteristics, there were no significant differences between the 53 mother/infant dyads who were excluded compared to the 50 mother/infant dyads who were included. There were no differences in maternal age, ethnicity, mode of delivery, percentage of primiparous women, or maternal weight gain between women with and without a history of allergic disease. There were significantly more women who smoked tobacco in the allergic group compared with the non-allergic group (35.7% vs. 5.6%, p = 0.014). No differences were noted in infant characteristics including birth weight, gestational age, sex, or 1- and 5-min Apgar scores.

**Table 1:** Maternal and neonatal characteristics based on maternal history of allergic disease

Baseline characteristics	Allergic moms (n = 14)	Non-allergic moms (n = 36)	P-value
Maternal characteristics			
Age, years			
Median (range)	33 (20–42)	33 (20–41)	1.00
Ethnicity, n (%)			
Caucasian	8 (57.2)	15 (41.7)	*0.36
Hispanic	4 (28.6)	11 (30.6)	
Black	1 (7.1)	3 (8.3)	
Other	1 (7.1)	7 (19.4)	
Mode of delivery, n (%)			
C-section	10 (71.4)	29 (80.6)	0.43
Primiparous, n (%)	5 (35.7)	9 (25.0)	0.50
Smoking, n (%)	5 (35.7)	2 (5.6)	0.014
Weight gain, n (%)			
10–20 lbs	3 (21.4)	4 (11.1)	0.48
21–30 lbs	6 (42.9)	13 (36.1)	
31–40 lbs	1 (7.1)	9 (25.0)	
> 40 lbs	4 (28.6)	10 (27.8)	
Neonatal characteristics			
Birth weight, grams			
Median (range)	3374 (2935–4875)	3581 (2331–4415)	0.83
Gestational Age, weeks			
Median (range)	39.3 (39.0–41.0)	39.2 (37.0–40.3)	0.11
Sex, n (%)			
Male	7 (50.0)	20 (55.6)	0.76
APGAR score n (%)			
@lmin ≤5	0	0	NS
@5min ≤7	0	0	NS

* p value is for Caucasian vs. non-Caucasian

### Serum IgE levels

**Figure 1:**
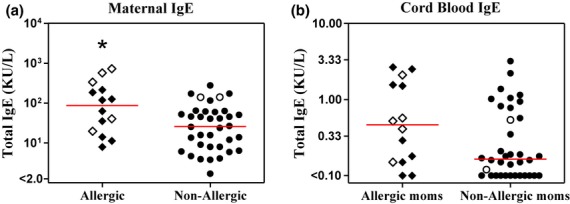
Total serum IgE levels based on maternal history of allergic disease. (a) Pregnant women with a history of allergic disease demonstrated significantly higher total serum IgE levels than pregnant women without a history of allergic disease. (b) Total serum IgE levels were not significantly different in cord blood samples obtained from infants of allergic mothers as compared to infants of non-allergic mothers. Open symbols indicate mothers or infants born to mothers who smoked tobacco during pregnancy. The horizontal line represents the median. *p = 0.03.

Pregnant women with a history of allergic disease demonstrated higher levels of serum total IgE than pregnant women without a history of allergic disease [median 90.95 (range 7.91–730.40) vs. 25.90 (1.70–276.50) KU/l, p = 0.03] (Fig. 1a). CB IgE levels were not significantly different between infants of allergic mothers and infants of non-allergic mothers [median 0.47 (range 0.10–2.67) vs. 0.17 (0.10–3.19) KU/l, p = 0.07] (Fig. 1b).

### Frequencies of CB basophils and pDCs

Frequencies of CB basophils and pDCs for the different groups of infants are shown in Table SI. There were no significant differences in frequencies of CB basophils in infants of allergic mothers compared with infants of non-allergic mothers [median 0.10 (range 0.03–0.75) vs. 0.13 (0.03–0.51), p = 0.47]. Similarly, there were no significant differences in frequencies of CB pDCs in infants of allergic mothers compared with infants of non-allergic mothers [median 0.37 (range 0.13–0.77) vs. 0.41 (0.09–2.28), p = 0.65].

### IgE and FcεRI expressions on CB basophils and pDCs

**Figure 2:**
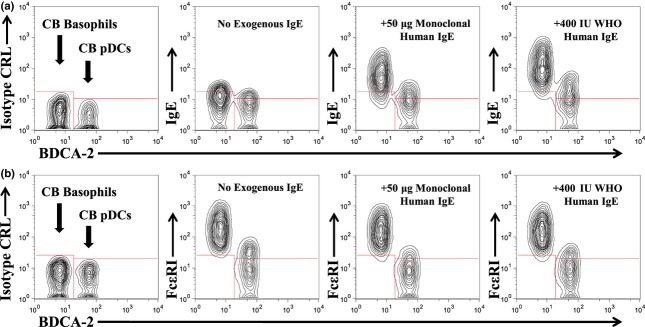
Identification of Cord blood (CB) basophils and pDCs, and quantification of surface-bound IgE and FcεRI expressions using flow cytometry. CB peripheral blood mononuclear cells (CBMCs) were pre-incubated with or without monoclonal human IgE (HE1) (50 μg) or polyclonal WHO IgE preparation (400IU) and then stained to identify basophils, pDCs, surface-bound IgE expression, and FcεRI expression. (a) CB basophils and pDCs that expressed low levels of endogenous surface-bound IgE were capable of binding exogenous IgE. (b) The binding of IgE to the surface of CB basophils and pDCs did not inhibit the ability to detect FcεRI. The percentages of cells staining positive for surface-bound IgE or FcεRI expression were quantified relative to an isotype control antibody.

We first determined the ability of CB basophils and pDCs to bind exogenous IgE. CBMC preparations were pre-incubated in the absence or presence of exogenous IgE. Following pre-incubation, cells were stained to identify CB basophils and pDCs, and to define each cell type’s capacity to bind IgE or level of FcεRI expressions. CB basophils and pDCs from a patient with low levels of endogenous surface-bound IgE were capable of binding monoclonal human IgE (HE1) or polyclonal WHO IgE (Fig. 2a). Furthermore, the binding of exogenous IgE to the surface of cells did not inhibit our ability to detect FcεRI (Fig. 2b).

**Figure 3:**
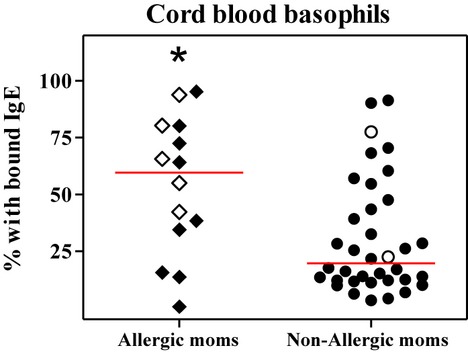
Percentages of Cord blood (CB) basophils with surface-bound IgE were significantly greater in infants of allergic mothers as compared to infants of non-allergic mothers. CB peripheral blood mononuclear cells (CBMCs) collected at the time of delivery were cryopreserved, thawed, and stained to identify basophils, pDCs, surface-bound IgE expression, and FcεRI expression. Flow cytometry was used to calculate the percentages of CB basophils with surface-bound IgE relative to an isotype control antibody. Open symbols indicate infants born to mothers who smoked tobacco during pregnancy. The horizontal line represents the median. *p = 0.01.

We next determined the percentages of CB basophils and pDCs with bound IgE or FcεRI expression from study subjects. The percentages of CB basophils with bound IgE were significantly greater in infants of allergic mothers compared with infants of non-allergic mothers [median 59.60 (range 0.69–95.40) vs. 19.70 (3.43–91.50)%, p = 0.01] (Fig. 3). There were no significant differences in percentages of CB pDCs with bound IgE in infants of allergic mothers compared with infants of non-allergic mothers [median 19.00 (range 2.45–59.60) vs. 15.20 (0.99–48.40)%, p = 0.27] (data not shown). Similarly, there were no significant differences in the percentages of CB basophils or pDCs expressing FcεRI in infants of allergic mothers compared with infants of non-allergic mothers [median 91.75 (range 72.70–98.20) vs. 89.50 (63.60–98.80)%, p = 0.93, respectively, for CB basophils or median 37.95 (range 8.56–71.50) vs. 42.35 (2.63–67.80)%, p = 0.86, respectively, for CB pDCs] (data not shown).

### Maternal and CB serum IgE levels and surface expression of IgE and FcεRI

**Figure 4:**
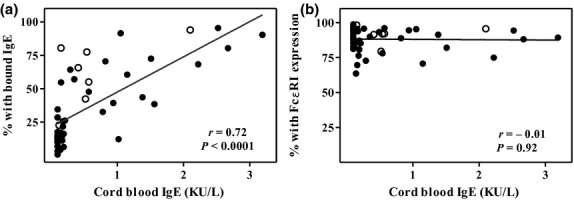
The relationship between Cord blood (CB) IgE levels and percentages of CB basophils with surface-bound IgE or FcεRI expression. Data from the allergic and non-allergic mother/infant dyads were grouped together, and the relationship was investigated using Pearson’s correlation. (a) Percentages of CB basophils with surface-bound IgE correlated strongly with CB IgE levels. (b) Percentages of CB basophils expressing FcεRI were not significantly associated with CB IgE levels. Open symbols indicate infants born to mothers who smoked tobacco during pregnancy.

To examine the association between maternal and CB serum IgE levels with bound IgE and FcεRI expressions by CB basophils or pDCs, data from the allergic and non-allergic mother/infant dyads were grouped together. There were no significant correlations between maternal serum IgE levels and percentages of CB basophils or pDCs with bound IgE (r = 0.26, p = 0.06 or r = 0.04, p = 0.76, respectively) (data not shown). In contrast, there was a significant correlation between CB IgE levels and percentages of CB basophils with bound IgE (r = 0.72, p < 0.0001; Fig. 4a). When examining for FcεRI expression, there were no significant correlations between maternal serum IgE levels and percentages of CB basophils or pDCs expressing FcεRI (r = −0.05, p = 0.75 or r = 0.27, p = 0.06, respectively) (data not shown). Similarly, there were no significant correlations between CB IgE levels and percentages of CB basophils or pDCs expressing FcεRI (r = −0.01, p = 0.92 or r = 0.10, p = 0.52, respectively) (Fig. 4b and data not shown).

**Figure 5:**
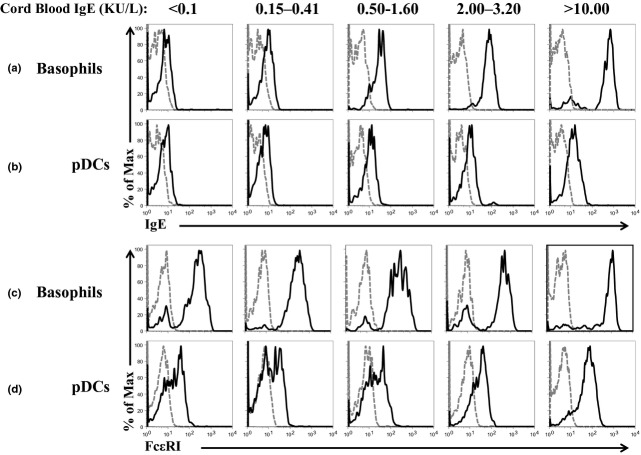
The relationship between the fluorescence intensities for bound IgE and increasing levels of Cord blood (CB) IgE. (a) CB basophils demonstrated increases in fluorescence intensities in parallel to increasing levels of CB IgE. (b) CB pDCs demonstrated minimal change. (c,d) CB basophils and pDCs demonstrated increases in fluorescence intensities for bound IgE over wide ranges of CB IgE levels. Data shown are representative of the typical staining profile in multiple CB cell specimens analyzed from patients with the indicated ranges of serum IgE. Dashed lines represent isotype controls.

In addition, there were increases in the fluorescence intensities for bound IgE detected on CB basophils, relative to an isotype control, in parallel to the increasing levels of CB IgE (Fig 5a). In contrast, minimal changes were observed in the fluorescence intensities for IgE detected on CB pDCs, relative to the increasing serum levels of CB IgE (Fig 5b). There were concomitant increases in fluorescence intensities for FcεRI detected on CB basophils and pDCs, over wide ranges of CB IgE levels (Fig. 5c,d).

## Discussion

In this study, we found that the frequencies of CB basophils and pDCs were similar in infants irrespective of whether they were born to mothers with or without a history of allergy that included symptomatic disease in the past 12 months. However, we also found that the percentages of CB basophils with surface-bound IgE were significantly greater in infants of allergic mothers compared with infants of non-allergic mothers. Our ability to quantify percentages of cells with bound IgE was possible because of the low serum IgE levels present in the majority of CB specimens. As CB serum IgE levels approached 2–3 KU/l, the percentages of cells loaded with IgE and detected using flow cytometry increased toward 100%.

Despite the apparent association between maternal allergy and IgE on CB basophils, we found no difference in CB serum IgE levels between infants born to allergic vs. non-allergic mothers. The reason for this discrepancy is unclear, but consistent with published reports on the poor concordance between maternal and CB IgE levels 20. It is known that IgE binds with high affinity to FcεRI on the surface of cells 21. Perhaps under conditions of low serum IgE, the ability to measure surface-bound IgE provides a more sensitive indicator of total IgE located within the fetal compartment 22–24.

Maternal IgG is generally regarded as the only antibody isotype capable of crossing the placental barrier 25. However, in mice, the neonatal Fc receptor for IgG uptake (FcRn) facilitates the intestinal absorption of maternal IgE in the form of IgG anti-IgE/IgE immune complexes 26. Furthermore, when IgG anti-IgE/IgE immune complexes are generated with IgG anti-IgE directed against the Cε4 domain, a region not involved in the binding of IgE to FcεRI, the IgE is biologically active, retaining the capacity to bind and induce allergen-specific β-hexosaminidase release from rat basophil leukemia cells 27. Because FcRn is expressed in the human placenta 28, it is possible that when pro-inflammatory conditions exist during pregnancy, this favors the generation and transmission of biologically active maternal IgG anti-IgE/IgE immune complexes to the fetus, with resultant binding to FcεRI-expressing fetal cells 29–32.

Because the overall numbers of CB basophils in our study were low, we were limited to investigating bound IgE and FcεRI expressions from the subset of samples containing sufficient cells for analysis. This also limited our ability to determine the functional consequences of IgE bound to the surface of CB cells. Basophils generated *in vitro* from CB mononuclear cells can be passively sensitized with IgE and release cytoplasmic granules upon challenge with anti-IgE or antigen 33, 34. In our study, we demonstrated that basophils isolated directly from CB specimens bound IgE determined by levels of IgE in CB serum. Taken together, these results suggest that fetal basophils may be functionally active *in vivo* and competent to participate in pre-natal or early post-natal allergic responses.

Recent studies demonstrate that a deficiency of circulating pDCs in early childhood is a risk factor for viral respiratory tract infections and allergic conditions such as asthma 15. In our study, we found that exposure to maternal allergy did not alter the frequencies of CB pDCs. Our results are in agreement with a previous study by Hagendorens et al. in which no difference in DC subtypes was detected between neonates at low versus high risk for allergic disorders 35. Our study expands these findings to more clearly define the relationship between maternal allergy and the subset of CB pDCs expressing the surface marker BDCA-2.

A potential limitation of this study was that significantly more women smoked tobacco in the allergic group compared with the non-allergic group. The low number of smokers in both groups limited our ability to do a stratified analysis. In addition, exclusion of smokers would have reduced the number of mother/infant dyads in the allergic group by 25% and potentially diminished the ability to detect statistically significant differences. The apparent relationship between maternal allergic disease and surface-bound IgE on CB basophils should therefore, be interpreted with caution. The distribution of smokers also appeared rather evenly distributed amongst the data suggesting that much larger numbers of smokers would be required to differentiate a potential effect. A second limitation was the high percentage of C-section births in both groups, which is a reflection of our convenience sampling as we found it more feasible to recruit women prior to scheduled C-sections as compared to vaginal deliveries.

In conclusion, this study demonstrates that frequencies of CB basophils and pDCs are not associated with maternal allergic disease. The finding of increased IgE on CB basophils from infants of allergic mothers suggests that cell-bound IgE may be a more sensitive indicator (than free IgE) of total IgE in the fetal compartment. Screening for cell-bound IgE, in addition to other parameters such as CB IgE 36 or CB T cell subsets 37, may improve the ability to identify babies at high risk of developing allergy.

## Supporting Information

Methods S1 Recruitment and eligibility criteria, Collection and preparation of cord blood cells for flow cytometry.

Figure S1 Flow diagram demonstrating numbers of allergic and non-allergic mother/infant dyads recruited, enrolled, and excluded. CB samples from 14 allergic mother/infant dyads and 36 non-allergic mother/infant dyads contained sufficient numbers of basophils and pDCs to evaluate the primary and secondary outcomes.

Table S1 Frequencies of CB basophils and pDCs identified in infants of allergic and non-allergic mothers. Frequencies of cells are expressed as percentages of total CBMCs.
