# Supplementary material for: Maternal allergy is associated with surface-bound IgE on cord blood basophils
Source: Pediatr Allergy Immunol. 2013 Aug 27;24(6):614–21. doi: 10.1111/pai.12113 (PMC3798094; doi:10.1111/pai.12113)
Supplement: Methods S1 — Recruitment and eligibility criteria, Collection and preparation of cord blood cells for flow cytometry. [file pai0024-0614-sd1.doc]

**Supplementary Methods:**

**Recruitment and eligibility criteria**

Allergic and non-allergic pregnant women receiving care at Hartford Hospital Associated OB/GYN practices (Hartford, Connecticut, USA) were informed of the study through an introductory letter included in prenatal information packets mailed to prospective parents. Upon admission to Labor and Delivery, potential participants were screened for eligibility and recruited for participation. Pregnant women were eligible if they were English or Spanish speaking, had not received prenatal steroids for the treatment of preterm labor, were not on high dose inhaled steroids (e.g. >800 mcg/day beclomethasone equivalent), and were delivering an infant ≥37 weeks’ gestational age with no known major congenital anomaly.

**Collection and preparation of cord blood cells for flow cytometry**

Cord blood samples were collected in CPT Cell Preparation Tubes with Sodium Citrate (BD, Franklin Lakes, New Jersey, USA) and kept at room temperature until processing which occurred within 24 h. Following centrifugation, cells were washed and suspended in 5% human albumin (Baxter Healthcare Corp., Westlake Village, California, USA) to achieve a concentration of approximately 12 x 10^6^ cells/ml. The CBMCs in 5% albumin were cooled to 4ºC and an equal volume of 20% DMSO (American Type Culture Collection, Manassas, VA) mixed with Dextran 40 (Protide Pharmaceutical Inc., Lake Zurich, IL), also cooled to 4ºC, was added to achieve a final concentration of approximately 6 x 10^6^ cells/ml. Cell suspensions were transferred to round bottom cryovials, brought to -80ºC in cryofreezing containers filled with 2-propranol, and then transferred to the gaseous phase of liquid nitrogen for long-term storage. When ready to be used, cryovials were removed from liquid nitrogen and transferred to a water bath at 37ºC. Thawed samples were suspended in Dextran/albumin (1:1) to 5 ml, centrifuged, and suspended in PBS containing 0.2% BSA and 0.1% NaN3 for staining. The viability of cells recovered after the thawing procedure was determined by tryptan blue exclusion and was 90-95%.

Cells were stained with the following mAbs: anti-BDCA-2/FITC (AC144), -CD123/PE (AC145), -IgE/APC (MB10-5C4) (Miltenyli Biotech Inc., Auburn, California, USA), -FcεRI/APC (AER-37) (eBiocience Inc., San Diego, California, USA), mouse IgG_1_ isotype control/APC (IS5-21F5) (Miltenyi Biotech), or mouse IgG_2b_ isotype control/APC (eBMG2b, eBioscience). After washing to remove unbound antibodies, stained cells were fixed in 3% paraformaldehyde, and relative fluorescence intensities were determined using a FACS Calibur (BD Biosciences, San Jose, California, USA). FlowJo software (Tree Star Inc., Ashland, Oregon, USA) was used to analyze data collected on the flow cytometer. The percentages of cells identified as “positive” for expression of IgE or FcεRI were based on the fraction of cells with fluorescence intensities greater than their respective isotype controls.

In some samples, the frequency of basophils and pDCs identified in cryopreserved cord blood specimens were compared with those identified in fresh cord blood samples. In cryopreserved specimens, the frequency of basophils was generally lower and the frequency of pDCs was generally higher than those observed in fresh samples, however the difference was not statistically significant (n=4). There were no differences in percentages of cord blood basophils or pDCs staining positive for IgE or FcεRI between cryopreserved or fresh specimens.

**Legends for Supplementary Figures and Tables:**

**Supplementary Figure 1:** Flow diagram demonstrating numbers of allergic and non-allergic mother/infant dyads recruited, enrolled, and excluded. CB samples from 14 allergic mother/infant dyads and 36 non-allergic mother/infant dyads contained sufficient numbers of basophils and pDCs to evaluate the primary and secondary outcomes.

**Supplementary Table 1:** Frequencies of CB basophils and pDCs identified in infants of allergic and non-allergic mothers. Frequencies of cells are expressed as percentages of total CBMCs.
