# Supplementary figures and images for: Maternal allergy is associated with surface-bound IgE on cord blood basophils
Source: Pediatr Allergy Immunol. 2013 Aug 27;24(6):614–21. doi: 10.1111/pai.12113 (PMC3798094; doi:10.1111/pai.12113)

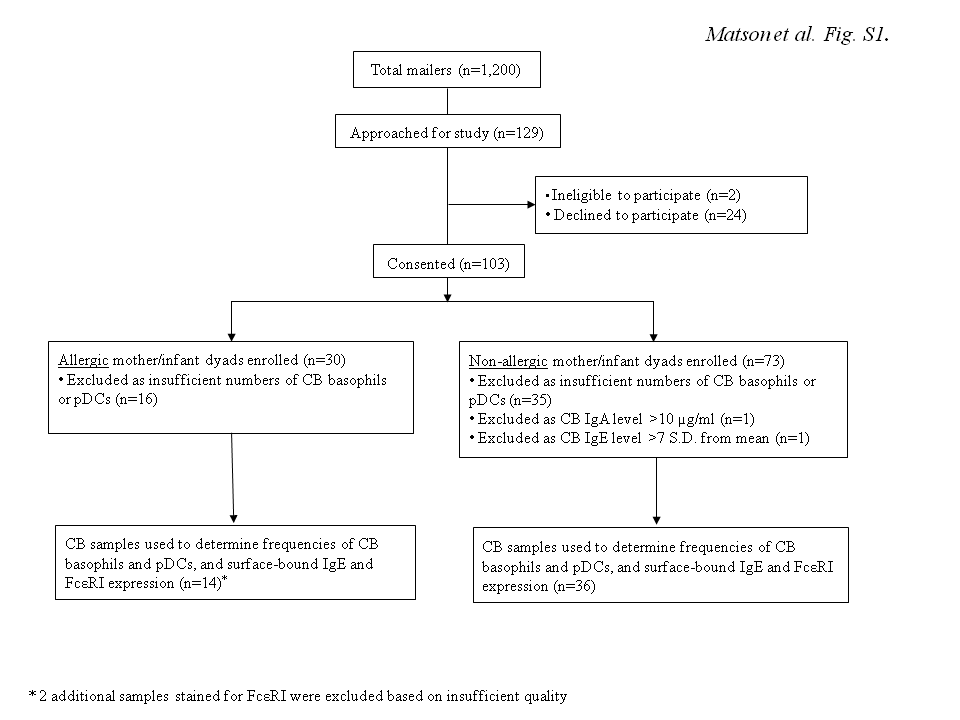

Supplement: Figure S1 — Flow diagram demonstrating numbers of allergic and non-allergic mother/infant dyads recruited, enrolled, and excluded. CB samples from 14 allergic mother/infant dyads and 36 non-allergic mother/infant dyads contained sufficient numbers of basophils and pDCs to evaluate the primary and secondary outcomes. [file pai0024-0614-sd2.tif]

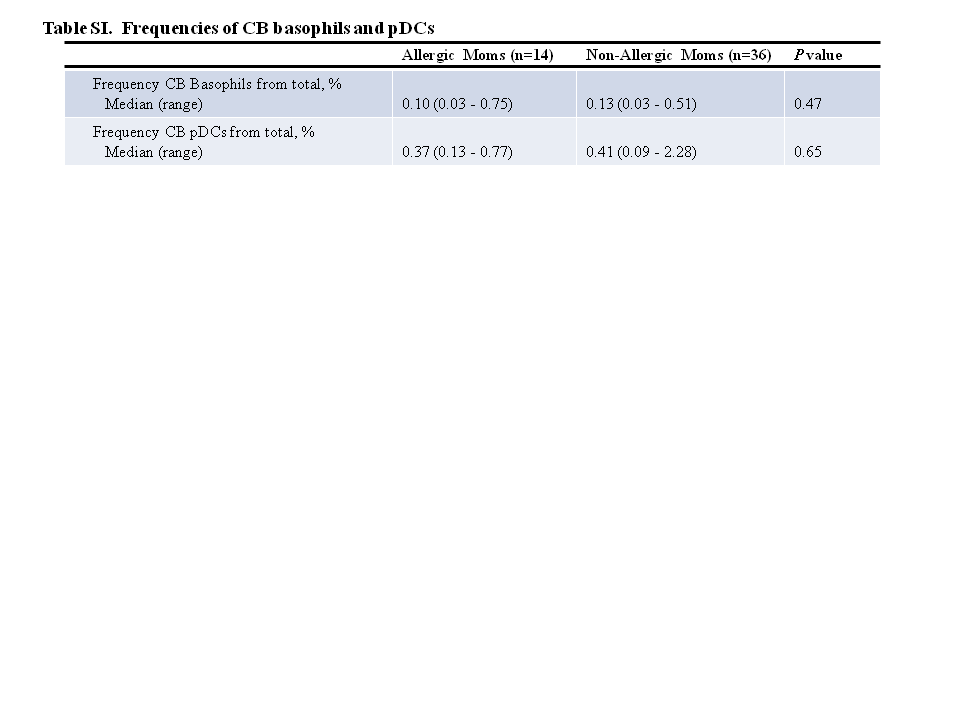

Supplement: Table S1 — Frequencies of CB basophils and pDCs identified in infants of allergic and non-allergic mothers. Frequencies of cells are expressed as percentages of total CBMCs. [file pai0024-0614-sd3.tif]
